# Successful Spinal Anesthesia in a Patient With Severe Coagulopathy From Acute Liver Disease of Pregnancy Undergoing Cesarean Section: A Case Report

**DOI:** 10.7759/cureus.98092

**Published:** 2025-11-29

**Authors:** Jingxia Meng, Shoaib Nawaz, Odai Khamash, Roni Mendonca, Ejaz Khan

**Affiliations:** 1 Anesthesiology, New York Medical College, Metropolitan Hospital Center, New York, USA; 2 Anesthesiology, UPMC (University of Pittsburgh Medical Center) Harrisburg Hospital, Harrisburg, USA; 3 Anaesthesiology, Metropolitan Hospital Center, New York, USA

**Keywords:** acute fatty liver of pregnancy, cesarean section, coagulopathy, neuraxial anesthesia, spinal hematoma

## Abstract

We present a case of acute fatty liver of pregnancy (AFLP) with significant coagulopathy despite no bleeding symptoms, which was managed with spinal anesthesia without adverse events. This phenomenon may not be attributable to coincidence alone. Upon reviewing the literature, standard coagulation tests fail to account for the simultaneous reduction in pro- and anticoagulant factors, leading to a misleading impression of coagulopathy and often overestimating the risk of bleeding. Thus, it is advised that coagulopathy tests associated with liver disease should not be used solely to dictate anesthesia decisions; a thorough history obtained from the patient, family, or medical records may be more informative than abnormal coagulation tests.

## Introduction

Coagulopathy refers to abnormalities in prothrombin time (PT)/international normalized ratio (INR) and activated partial thromboplastin time (aPTT). The aPTT is a screening test for deficiencies or inhibitors affecting the intrinsic (Factors VIII, IX, XI, XII) and common (Factors II, V, X, fibrinogen) coagulation pathways. PT screens for abnormalities in the extrinsic pathway, and the INR standardizes PT results across different laboratories.

Acute fatty liver of pregnancy (AFLP) is a rare, life-threatening pregnancy complication that typically arises in the third trimester or postpartum period and requires prompt diagnosis and intervention. Due to associated liver dysfunction, AFLP patients often exhibit laboratory coagulopathy. Current guidelines from the American Society of Regional Anesthesia and Pain Medicine (ASRA) recommend an INR of ≤1.4 for safe neuraxial block placement as the potential bleeding risk and hematomas, especially spinal epidural hematoma [1].

However, emerging evidence over the past two decades challenges the notion that liver diseases result in an acquired bleeding disorder. Some studies even suggest an increased risk of venous thrombosis in patients with liver disease [2]. Nonetheless, coagulopathy is frequently regarded as a contraindication to invasive procedures in our anesthesia practice. This case of AFLP highlights the safe use of spinal anesthesia, questioning the conventional dependence on coagulation parameters in anesthesia planning.

## Case presentation

A 41-year-old female, G10P3063, at 38 weeks of gestation, presented with nausea, vomiting, and heartburn. On admission, her blood pressure was 140/109 mmHg, and her heart rate was 116 bpm. The initial physical examination was unremarkable. However, fetal monitoring revealed recurrent late decelerations with minimal variability, prompting an urgent cesarean delivery. Due to the emergent nature of the case, there was insufficient time to await lab results.

Given the patient’s active vomiting, spinal anesthesia was administered using 1.6 mL of hyperbaric bupivacaine (0.75%, 12 mg). Shortly after the procedure, icteric sclerae were noted. A stillborn male fetus was delivered. The anesthesia team promptly placed an additional large-bore intravenous (IV) line in anticipation of a possible blood transfusion. Estimated blood loss was 1300 mL, and 350 mL of blood was transfused. Emergent lab tests, including liver function tests (LFTs) and coagulation profiles, were obtained, and close neurological monitoring was initiated to assess for potential spinal or epidural hematoma.

**Figure 1:**
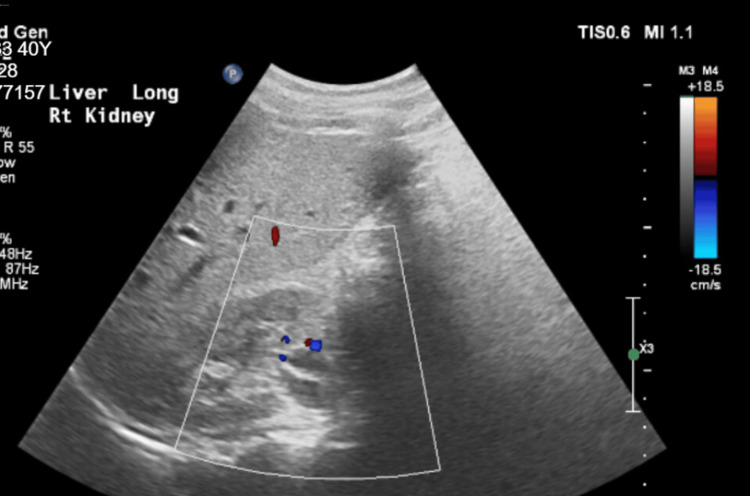
Liver ultrasound

Laboratory results revealed transaminitis, prolonged PT/INR and aPTT, acute kidney injury, and hypoglycemia, confirming the diagnosis of AFLP based on the Swansea criteria. The liver ultrasound showed a bright echogenic liver, which is indicative of diffuse fatty liver infiltration (Figure 1). No spinal or epidural hematoma developed.

On postoperative day 4, the patient’s hemoglobin dropped to 6.4 g/dL, platelets decreased to 44 × 10⁹/L, and a hematoma developed within the abdominal rectus muscles. Dynamic lab test results are shown in Table 1.

**Table 1:** Laboratory test results (baseline labs were done seven months ago) AST: aspartate aminotransferase; ALT: alanine aminotransferase; ALK PHOS: alkaline phosphatase; WBC: white blood cell; LDH: lactate dehydrogenase; aPTT: activated partial thromboplastin time; PT: prothrombin time; INR: international normalized ratio

	Baseline	admission	Day 2-6 Lowest/highest	Day 7	Day 10	Normal reference value
AST	21 (7 months ago)	532	532	70	49	0 - 32 U/L
ALT	29	611	611	29	31	0 - 31 U/L
ALK PHOS	87	545	545	94	114	35 - 104 U/L
Total/direct bilirubin	0.4/NA	6.0/4.0	10.2/6.6	7.9/4.6	5.9/2.8	Total Bilirubin 0.0 - 1.2 mg/dL, Direct Bilirubin 0.0 - 0.3 mg/dL
Total protein/albumin	7.1/4.4	7.0/3.8	5.1/2.0	5.3/3.5	7.1/5.3	Total Protein 6.6 - 8.7 g/dL, Albumin 3.5 - 5.2 mg/dL
Creatinine	0.67	3.7	6.8	2.2	1.1	0.7 - 0.9 mg/dL
Hemoglobin	14.1	17.9	6.4	9.0	11.1	12.0 - 16.0 g/dL
Platelet	313	188	44	44	146	150 - 450 x10(3)/mcL
aPTT	N/A	53.6	53.6	30.7	26.7	25.1 - 36.5 seconds
PT/INR	N/A	22.6/2.1	22.6/2.1	14.1/1.2	12/1	PT 9.4 - 12.5 seconds INR 0.9-1.1
WBC	9.28	19.7	30.7	10.8	12	4.30 - 11.00 x10(3)/mcL
Glucose	90	50	50	100	158	74 - 109 mg/dL
Fibrinogen	N/A	125	75	194	195	200 - 393 mg/dL
Haptoglobin	<20	<20	<20	<20	<20	34 - 200 mg/dL
LDH	N/A	595	814	814	659	135-214 U/L
Ammonia	N/A	75	90	35	27	16-33 mmol/L

Over the following days, she developed pancreatitis, disseminated intravascular coagulation (DIC), and renal failure. Supportive care was provided, and she was discharged on postoperative day 13 in stable condition.

## Discussion

Our patient met Swansea criteria for AFLP, including coagulopathy, gastrointestinal symptoms, hypoglycemia, renal impairment, and leukocytosis. Spinal anesthesia was selected based on clinical presentation - vomiting and the absence of bleeding symptoms - despite later discovery of significant coagulopathy. Most AFLP-associated cesarean deliveries reported in the literature are performed under general anesthesia or under neuraxial anesthesia after INR correction (to <1.5) or administration of blood products, vitamin K, or antifibrinolytics [3-5]. Our case contributes to the evolving discussion on whether abnormal coagulation tests should uniformly preclude neuraxial anesthesia.

The principal concern with neuraxial anesthesia in coagulopathy is spinal epidural hematoma (SEH). SEH has been reported in the context of anticoagulant or thrombolytic therapy; however, there is scant evidence linking liver disease-associated coagulopathy to SEH. To our knowledge, only one case report from 1978 describes SEH in this context [6], and there are no reports involving pregnancy-associated liver disease. It is worth noting that our patient did not develop a spinal epidural hematoma; however, she later developed a rectus sheath hematoma at a time when coagulation studies were normal but platelet counts had reached their nadir. This observation further underscores the weak correlation between laboratory-defined coagulopathy and actual bleeding risk.

Pregnancy itself induces a hypercoagulable state, and younger obstetric patients likely have a larger, more compliant epidural space compared to elderly patients. A recent study of 79,837 peripartum patients found no recognized cases of SEH [7]. Conversely, SEH can occur even with normal coagulation profiles. Cases include a child with sickle cell disease developing a thoracic hematoma post-epidural placement despite normal labs [8], and a review of 613 SEH cases found that nearly one-third had no identifiable risk factors [9].

Historically, liver disease was thought to create a bleeding diathesis. However, studies show that standard coagulation tests poorly correlate with bleeding risk in liver disease [10] and may even reflect a procoagulant state [11]. The liver regulates primary hemostasis, coagulation, and fibrinolysis, and dysfunction affects both procoagulants and anticoagulants, maintaining a balance in the coagulation system [12]. Provided the platelet count is adequate, patients with liver disease can continue to generate thrombin and fibrin. A Danish population-based cohort study demonstrated a low absolute risk of spinal hematoma within 30 days of lumbar puncture, with no increased risk in patients with coagulopathy [13].

An interdisciplinary task force (including members from the American Society of Regional Anesthesia and Pain Medicine (ASRA), American College of Obstetricians and Gynecologists (ACOG), Society for Maternal-Fetal Medicine (SMFM), and American Society of Hematology (ASH)) concluded that PT and aPTT offer limited value in assessing the safety of neuraxial anesthesia for obstetric patients. Instead, family history and bleeding history (such as post-surgical bleeding) are considered more informative [14].

Our case report has limitations. First, it does not apply to the coagulopathy after massive hemorrhage, and may not apply to patients with other comorbidities such as immunosuppressed patients or those on anticoagulation. Second, our patient had normal platelet counts at the time of spinal anesthesia. Third, larger prospective or retrospective cohort studies are needed to better quantify the bleeding risk. Finally, regardless of laboratory values, vigilant postoperative neurological monitoring remains essential.

## Conclusions

This case report and literature review suggest that liver disease-associated coagulopathy may not substantially increase the risk of bleeding or spinal epidural hematoma and, therefore, should not automatically preclude neuraxial anesthesia. Reliance solely on conventional coagulation tests may be misleading and could result in unnecessary delays in patient care. A careful clinical history and physical examination remain essential.

For general anesthesiologists, this case provides practical insight into safely managing parturients with abnormal coagulation profiles, particularly in urgent or unexpected obstetric situations where subspecialty consultation may not be immediately available. For obstetric anesthesiologists, it reinforces the importance of individualized, physiology-based assessment and supports the selective use of neuraxial anesthesia in patients with liver disease when other bleeding risk factors are absent. Together, these perspectives highlight the need to re-evaluate current guidelines and to critically appraise the predictive value of standard coagulation assays in assessing bleeding risk.
